# Applied Research on the Effect of Risks on Public Health Building Projects′ Performance: Empirical Results From Tanzania

**DOI:** 10.1155/tswj/2617627

**Published:** 2026-06-26

**Authors:** Ismail W. R. Taifa, Kimy Suphian Massasi

**Affiliations:** ^1^ Department of Mechanical and Industrial Engineering, College of Engineering and Technology, University of Dar es Salaam, Dar es Salaam, Tanzania, udsm.ac.tz

**Keywords:** Dar es Salaam, project performance, public health-building, public health facilities, risk management practices

## Abstract

Public health building projects encounter several risks throughout the project phases. Using a quantitative research approach, the study assessed risks, the effects of risks encountered in public health building projects and proposed risk management practices to enhance project performance. Fifty‐six projects were involved in the present study. SPSS 21.0 and Microsoft Excel 2021 were used to analyse data, including regression analysis, linearity test, and risk heat map development. The overall Cronbach′s alpha for the collected data was 0.809, indicating that the data were reliable. The findings show that most risks were at a low level, leaving fewer at the medium level, including the contractor′s delay in cash flow, price fluctuations due to inflation and pollution from construction waste. The risk heat map visualised all risks by indicating each identified risk′s severity (impact) and likelihood (occurrence). Furthermore, the regression model for the effect of the assessed risks on the performance of public health‐buildings showed that there was a negative effect on the performance for all the measured risks (technological risks, financial risks, design risks, client or owner risks, management risks, construction risks, political risks, environmental risks, procurement risks and other risks). However, for the significance of those effects, the analysis demonstrated greater significance (i.e., *p* < 0.001) for two categories of risk: financial risks and client/owners′ risks. Subsequently, there was a significant effect on the performance of public health building projects from the other six categories of risks (technological risks [*p =* 0.041], design risks (*p =* 0.031), management risks (*p =* 0.012), construction risks (*p =* 0.024), environmental risks (*p =* 0.049) and procurement risks (*p =* 0.047). The effects and significance of the risk categories on each public building project performance indicator (cost, schedule and time) are presented using unstandardised beta coefficients and *p* values. In conclusion, the study has demonstrated that, despite the many risks and risk factors analysed in public health projects being rated at low to medium levels, they still exert a statistically significant negative influence on project performance—particularly financial and client‐related risks—therefore necessitating continuous monitoring and proactive management. The adoption of structured and adaptive risk management practices is therefore essential to mitigate potential impacts and enhance project outcomes in terms of cost, time and quality.

## 1. Background

Managing risks in public healthcare facilities is critical for ensuring patient safety, operational efficiency and the sustainability of healthcare services [1]. Risk management practices encompass strategies to identify, assess and mitigate potential hazards that could adversely affect the quality of care and the safety of patients and staff. International consensus indicates that successful risk governance remains a prerequisite for enduring performance in any healthcare setting. In response, national systems have progressively embraced multidimensional risk management infrastructures designed to simultaneously mitigate clinical, operational, financial and ecological exposures. Such infrastructures are operationalised through recurring risk appraisals, mandatory standard operating procedures and frameworks for iterative reassessment and enhancement of risk‐control measures [2]. The strategic diffusion of digital solutions, notably integrated electronic health records (EHRs) and centralised incident‐reporting platforms, has accelerated prospective and concurrent risk surveillance. Equally, the deliberate cultivation of transparent safety cultures, in which personnel are openly invited to disclose near‐misses and harm events without disciplinary repercussion, remains a decisive enabler for the advancement of patient‐centred safety [3].

Within high‐income nations, the United States serves as a pertinent case exemplifying the systematic integration of risk management within health systems. The Agency for Healthcare Research and Quality (AHRQ) has produced a suite of structured instruments and informational resources intended to guide providers in the practical application of risk mitigation. Central to the AHRQ′s approach is the alignment of executive leadership, proactive participation of clinical and administrative staff and the leveraged application of data analytics to uncover and neutralise emerging threats [4]. The National Health Service (NHS) in the United Kingdom has implemented a unified risk management framework that includes structured risk assessment matrices, standardised incident‐reporting channels and evidence‐based clinical governance directives [5]. This approach is replicated at the national level. In the interim, healthcare facilities have begun to formalise risk governance by adopting internationally recognised occupational health and safety standards, specifically International Organization for Standardisation (ISO) 31000 and ISO 45001. This is occurring within a broader trans‐European context. Research evidence suggests that hospitals that have implemented these models in clinical practice have experienced significant improvements in three metrics: the volume and quality of incident disclosures, improvements in quantifiable patient safety metrics and increased participation of clinical staff in risk mitigation initiatives [6]. Collectively, these regulations establish a requirement for inclusive engagement across all stakeholder strata within the risk management continuum, in addition to mandating iterative advancement.

There are numerous continent‐specific constraints that impede the incorporation of systematic risk management in African public healthcare settings, including a persistent deficit of qualified personnel, underdeveloped physical infrastructure and limited fiscal capacity [3]. The ability of healthcare facilities to identify, assess and mitigate risks in a timely and coordinated manner is diminished by these constraints. However, clear improvements in risk governance are apparent in a variety of sub‐Saharan contexts. The African Partnerships for Patient Safety (APPS), a World Health Organisation (WHO)‐hosted initiative, has facilitated the dissemination of safety and risk management protocols by organising, harmonising and customising interventions to various national healthcare ecosystems. In addition to this continental endeavour, Kumah [7] notes that Ghana has implemented a phased risk management strategy characterised by the systematic codification of clinical protocols into standard operating procedures, the establishment of multidisciplinary infection control committees and targeted competency‐based training for clinical and administrative staff. This strategy institutionalises the continuous monitoring and control of clinical hazards.

Recent efforts in Nigeria to improve risk management practices have focused on the dual objectives of improving the training of healthcare personnel and strengthening health information systems. The strategic incorporation of electronic health information systems, as demonstrated by the research conducted by Adepoju and Esan [3], facilitates the opportune detection of emergent clinical hazards by enabling more precise monitoring of patient records. Parallel to this, the systematic implementation of continuous professional development initiatives has increased risk awareness and improved staff competencies. A similar approach is demonstrated in the Republic of South Africa, where the National Department of Health has incorporated risk management into the National Core Standards for health facilities. The South African National Department of Health [8] set forth these standards, which include specific criteria for the governance of clinical hazards in healthcare environments. By requiring the active involvement of facility leadership, the regular implementation of staff training and periodic execution of conformance audits, the framework establishes a structured mechanism for consistent improvement in patient safety benchmarks.

Across Tanzania, attention to formalised risk management within public healthcare institutions has accelerated, driven by an overarching ambition to strengthen the nation′s medical delivery system. Region‐wide, the Tanzanian system mirrors the resource constraints and infrastructural bottlenecks confronted by many other African nations [9]. In response, projects targeting the enhancement of risk management habits have already commenced. Haule and Barongo [10] report that health institutions are progressively operationalising risk management blueprints, manifesting in recurrent risk evaluations, the enactment of safety protocols and the capacitation of personnel to recognise and counter threats. Their investigation further indicates that technological resources—most notably health information platforms—are being incrementally woven into these risk‐oriented practices, even as infrastructural shortfalls and insufficient preparatory training impede seamless integration.

In Tanzania, the finalisation of public healthcare facility completion and the concomitant construction of new facilities mandate finely calibrated risk management strategies. Phoya [11] delineates that the construction phase itself harbours a suite of discrete and interconnected risks, chief among them schedule slippage, cost escalation and occupational safety events. Following the defect liability period, effective management of analogous risks remains imperative to ensure the intended operational state and safety of the asset. An integrated risk management framework for healthcare buildings at hand encompasses the mitigation of clinical and operational risks while concurrently affirming the facility′s structural capacity and overall functionality. Within a supportive regulatory regime, Sospeter and Chileshe [12] cite directives issued by the Tanzanian Ministry of Health, which concentrate formal oversight on construction and life cycle operation processes, thus institutionalising systematic risk management practice.

The World Health Organization has formulated a global risk management framework for healthcare settings, the tenets of which Tanzanian authorities and implementing agencies may profitably adapt [13]. The framework advances a tripartite schema constituted by risk appraisal, risk amelioration and persistent surveillance. Central to this paradigm is the promotion of a pervasive safety culture, a mechanism predicated on the voluntary engagement of all clinical and ancillary personnel, reinforced by demonstrable, sustained support from institutional leadership [13].

Effective risk management in healthcare settings requires a cohesive strategy grounded in well‐articulated policies, transparent communication pathways and ongoing workforce education [14]. Enhanced clinical outcomes and a decrease in preventable damage are associated with extensive staff involvement in risk mitigation tasks, as evidenced by empirical evidence. Additionally, devoting priority instruction to risk management fosters a safety culture that is resilient and contributes to overall operational efficiency [7].

Within Dar es Salaam′s public health infrastructure, facilities serve as cornerstones for the delivery of essential services. Nevertheless, their capacity to function reliably is undermined by a constellation of risk management obstacles, such as chronic budget shortfalls, protracted project execution, inconsistent quality oversight and chronic administrative inefficiencies. Although a spectrum of established risk management measures—comprising risk identification, systematic appraisal, the formulation of mitigation directives and ongoing surveillance—has been theorised, residual vulnerabilities remain inadequately contained. Standard protocols include structured risk identification lists, risk scoring and prioritisation, task‐specific mitigation plans, ongoing situational surveys, clinical staff education seminars, compliance audits and enforced adherence to established clinical practice guidelines. Despite these preventive structures, the cumulative incapacity of the system to staunch recurrent operational lapses persistently diminishes the performance and viability of the facilities.

Notwithstanding the actionable frameworks that have been adopted, persistent deficiencies undermine the operational effectiveness of risk management protocols in public health infrastructure. Zhou [15] enumerates four principal shortcomings: the prevailing risk assessment methodologies fail to achieve normative rigour, there is an observable deficiency in the continuity of risk surveillance, personnel training remains sporadic and active contribution of stakeholder cohorts is sporadically institutionalised. Equally critical is the limited integration of risk management into the overarching governance fabric of public health installations [15]. The absence of harmonised protocols and toolbox instruments consequently impedes the transfer of validated best practices from one facility to another. This inquiry consequently seeks, through an empirical appraisal of concluded public health investments in Dar es Salaam, to determine the prevailing risk management practices, distil the impediments to their implementation and elucidate decisional pathways that solicit wide, durable conformity.

The study′s main objective is to assess the effect of risks on the performance of public health building projects in Dar es Salaam. Specifically, the study achieved the following research questions: (i) What risks are encountered in completed public health building projects in Dar es Salaam? (ii) How do risks affect the performance of completed public health building projects in Dar es Salaam? (iii) What are the risk management practices for the public health building projects in Dar es Salaam?

## 2. Literature Review

### 2.1. Risk and Risk Management

Chileshe and Kikwasi [16] characterise risk as any action or event that could impede the achievement of project objectives. They note that risks and uncertainties in the building industry surpass those in other sectors [16]. However, Hillson′s [17] definition of risk is adopted for this study. Hillson [17] defines risk as the potential occurrence of an event or circumstance that may positively or negatively impact the project′s objectives. Project risk encompasses uncertainties that, if materialised, could influence the project′s schedule, budget, quality or overall success. Risk management is a systematic process that entails identifying, assessing, prioritising and mitigating risks to achieve organisational objectives [18]. It involves recognising potential uncertainties, analysing their potential impacts and devising and executing strategies to manage or respond to these uncertainties. For this study, the definition of risk management by Wideman [19] is adopted, which characterises risk management as involving the identification, assessment and prioritisation of uncertainties, followed by the coordinated and efficient allocation of resources to minimise, control and monitor the impact of such uncertainties.

### 2.2. Public Health Facility

Garcia et al. [20] defined a health facility as any site, institution or structure that provides health services to people or communities. Health facilities come in a wide range of sizes and types, from tiny clinics and doctor′s offices to huge hospitals and speciality medical institutes. Health facilities are places that provide health services, such as hospitals, health centres and clinics. These places may provide a range of services, including primary care, specialised care, emergency treatment and rehabilitation. Public health facilities are healthcare facilities that the government pays for and runs in order to improve the health of the public by providing both preventative and curative treatments [21]. Gulliford et al. [22] report that a public health facility is a government‐run or publicly financed place that provides health services and improves the health of the community or area. These places usually provide a wide range of services, including health education, immunisation programs, treatment for illnesses and accidents and preventative care.

### 2.3. Public Health Building Projects

Building public health initiatives aims to enhance the health and well‐being of communities via infrastructure, services and policies. Some examples are building new facilities and health education programs; making existing facilities better; making public health policy; doing more research on new technologies; starting immunisation programmes; setting up centres for mental health, counselling services and support groups; doing environmental and community health projects; pushing for good nutrition and healthy eating; and doing comprehensive community health surveys [23]. These initiatives are meant to make healthcare more accessible, improve sanitation and clean water systems and housing conditions, and encourage people to live healthier lives. Also, these programs focus on research and development to enhance public health in both technical and strategic ways. Mental health initiatives point to the establishment of mental health centres, counselling services and support groups that cater to specific mental health needs. Environmental health projects aim to conserve the environment and eliminate hazards associated with health difficulties. Advocacy and education campaigns on nutrition and healthy eating aim to eliminate food insecurity and malnutrition.

Health building encompasses the construction, renovation and modernisation of hospitals, clinics and other centres. The project′s focus is on developing secure, orderly spaces that are conducive to patient care and the medical staff′s needs. These environments need infection control measures, positive healthcare physical environments to reduce stress and advanced medical technology [24]. Some specific examples include constructing new hospitals, renovating and expanding them and constructing clinics, medical offices and specialised centres. Other key characteristics include sustainable, modern frameworks, accessibility, eco‐friendly, positive healthcare environments, infection control, modular and prefabricated construction and specialised centres. Health building projects are essential to improve the standards of patient care and optimise staff performance, social benefits and overall public health. Usable facilities increase the rate of positive outcomes, decrease hospital readmissions and increase overall satisfaction with healthcare services. Such facilities expand their service areas and create employment opportunities, thereby supporting economic growth. Moreover, such facilities help improve public health by limiting the spread of infections and by providing access to quality health services through properly designed and maintained spaces.

### 2.4. Theoretical Literature

Theoretically, this study is guided by institutional theory, which examines the influence of social institutions on organisational behaviour and practices, and by resource dependency theory (RDT), which focuses on how organisations depend on external resources for survival and success.

#### 2.4.1. Institutional Theory

Institutional theory, first proposed by Meyer and Rowan [25], offers a comprehensive framework for examining how organisations respond to various institutional constraints, a phenomenon that is especially prominent in the implementation of public construction projects. Meyer and Rowan categorise these pressures into two interdependent dimensions: official sources, including regulatory frameworks and industrial standards, and informal sources, including cultural templates and normative society expectations. This bifurcation demonstrates that, within the realm of public health infrastructure, the arrangement of risk management practices by project stakeholders cannot be fully comprehended through internal decision‐making logics alone; it is significantly influenced by existing institutional scripts. The three external structures that make up the framework for these practices are following industry standards, complying with government laws and adhering to cultural norms for reducing risk. The theoretical agenda becomes clearer when we look at how organisations in the building industry act on a daily basis. Following risk assessment protocols, following health and safety rules and strategically taking part in public stakeholder engagement procedures are all signs of institutional pressures. These regulatory changes, which are also made to make them more effective, are always chosen for their intrinsic and competitive credibility. This makes adherence a quasinormative performance criterion that boosts legitimacy in the eyes of many different groups. Despite these analytical yields, the theory is limited; its explanatory power wanes in circumstances characterised by divergent constraints or when firms intentionally adopt disconnected practices to alleviate the cognitive load imposed by formal compliance.

Critics argue that blindly following institutional norms may lead to isomorphic conduct, when organisations prioritise ritual conformity and benchmarking above real effectiveness. In the field of risk management, this means focusing too much on meeting set audit schedules and compliance checklists, which often means not dealing with the site‐specific and quickly changing risk environment that is typical of public health delivery systems. Furthermore, the cumulative impact of institutional expectations can stifle innovative potential and adaptive foresight, forcing organisations to abandon strategic deviation in favour of the reassuring routine of established practices, even when alternative risk management paradigms demonstrate superior temporal or contextual logics.

#### 2.4.2. RDT

RDT, formally articulated by Jeffrey Pfeffer and Gerald R. Salancik, provides a lens through which to scrutinise the transactional and contingent bonds that condition access to, and stewardship of, critical resources in modern organisations [26]. RDT posits that organisations actively construct and reconstitute boundaries to attenuate vulnerability to a single locus of dependency. This is achieved through the strategic mobilisation of external inputs, which encompass financial capital, domain‐specific intelligence, proprietary or commercial technology, essential physical inputs and the tacit or active endorsement of salient stakeholder constituencies. To illustrate, public health delivery institutions systematically cultivate a web of alliances that connect them to pharmaceutical distributors, regulatory ministries, budgetary sponsors and the resident populace, each link serving to buffer the organisation against fluctuating supply chain exigencies, compliance uncertainty, fiscal stringency and the tacit or overt contestation of their clinical mission.

RDT helps public health organisations manage external dependencies by negotiating strategic alliances with suppliers, cultivating stakeholder linkages and securing an enduring rapport with regulatory authorities. However, RDT requires a comprehensive inventory of all external dependencies, which is challenging due to the complexity of the environment. Institutional theory explains how public health organisations align their structures with external norms, such as regulatory edicts and profession‐wide standards. Risk management efficacy is evaluated based on the organisation′s congruence with external demands, prevalence of imitative practices and responsiveness to coercive and normative influences. RDT highlights the importance of capital, human competence and civic endorsement in healthcare organisations. Such a standing, in turn, diminishes the prospect of premature resource retraction or overt dissent originating from these same entities. The observed consolidation of credibility within the construction arena thus echoes the prescriptive arguments of RDT, which accords central importance to calibrated management of external dependencies as a precondition for the ongoing viability and strategic success of institutions [27].

### 2.5. Empirical Literatures

#### 2.5.1. Risks in Public Health Building Projects

Even after completion, public health facilities confront an array of residual risks that jeopardise operational continuity, safety and service delivery. Xu et al. [28] identify human resource–related vulnerabilities as dominantly operational. Many institutions show chronic shortages of personnel, insufficient training and elevated attrition, each of which erodes continuity of care and inflates running expenses [28]. Hardware vulnerabilities, fiscal pressure, regulatory volatility, environmental contingencies, technological intrusion and infrastructural decay contribute to operational fragility in health facilities. Tight budgets, declining service standards and environmental forces increase risk. Digital systems pose cyber threats, and neglected physical structures necessitate sustained renewal. A multidimensional approach, including preventive maintenance, staff retention strategies and adaptive planning, is crucial for maintaining operational integrity.

According to Soltanzadeh et al. [2], construction projects are intricate and dangerous workplaces, especially regarding the sustainability of development, growth and worker risks, including environmental dangers. Other factors, such as the number of workers participating in the project, the methods and techniques of construction, the complexity of operations, the use of heavy plants as well as equipment and materials, multi‐interface operations and different types of workforce with divergent disciplines, contribute to the challenges facing the sector [29]. These factors contribute to increased accidents, including but not limited to falls from height, collisions, collapse and electric shocks [30]. More than any of the other construction sectors, the construction industry is regarded as one of the world′s most incident‐prone and hazardous construction occupations. Moreover, 25%–50% of other catastrophic and fatal incidents in industrialised countries are related to construction activities. Countries such as the United States, South Korea and China maintain high fatal occupational injuries, specifically ‘fell from a higher level’ and ‘struck by an object’ accident types [2]. Construction project occupational incidents, such as falls, slips and throws, abrasions and collisions, present direct and indirect costs, resulting in adverse social consequences of legal prosecution, damage to organisational reputation and a decline in project workmanship standards. These factors, if considered, can greatly enhance the productivity of the said organisations [2]. Constructive undertakings of public health projects encompass many risks, such as patient safety, worker safety and facility safety. These risks involve construction hazards, including slips and falls, asbestos exposure and dust and noise exposure—management compromises regarding funding and timeliness also present risks to the safety and successful completion of the project.

According to Soltanzadeh et al. [2], considerable attention is given to ensuring the workers′ safety due to construction site hazards, including dust, noise, exposure to hazardous substances and falls and slips. Electrical manual handling, noise and air contamination are also risks. Construction increases the risk of other infections, disrupting infection control. Attention has also been directed towards patient safety, where construction presents risks, including falls, as well as exposure to dust and noise. There are also risks associated with loss of operations in regard to a reduction in the period during which patient care and emergency services are available. Uncontrolled delays can negatively impact patient care and crucial facility building. Changes in delay design within the project also present risks in regard to cost overruns and completion challenges, hence affecting project integrity. Budget cuts offered by inadequate management pose risks to safety, causing overspending and delays. Compromised funding leads to limited scope, quality and timeliness of project completion [2].

#### 2.5.2. Risk Management Practices Employed in Public Health Facilities

Effective risk management practices in completed public health facilities are critical for safeguarding patient safety, enhancing operational efficiency and upholding the quality of clinical care. The core of such practices lies in establishing a comprehensive risk management framework [31]. This structured approach mandates the methodical identification, evaluation and ranking of risks, followed by coordinated actions designed to mitigate, track and govern the likelihood or consequence of adverse occurrences. Complementing this framework, the appointment of a dedicated risk management team—advocated by Khan [32]—ensures ongoing surveillance, delegated authority and sustained accountability for the execution of risk management tasks.

Continuous education and training of the clinical and operational workforce constitute a decisive component of risk governance [31]. Targeted training initiatives convey current risk management methodologies and competencies to staff and may take the form of workshops, seminars and realistic simulation exercises. Such pedagogical strategies equip personnel to respond effectively to a spectrum of anticipated hazards. Furthermore, a robust risk communication strategy—illuminated by Covello and Sandman [33]—necessitates the prompt, transparent and comprehensive dissemination of risk‐related information among all stakeholders, including employees, patients and the broader community. Finally, the execution of periodic audits and systematic inspections provides an essential mechanism for the continuous verification and enhancement of risk management standards, thereby sustaining an environment in which patient and operational safety are vigilantly maintained.

Routine financial audits, combined with systematic operational inspections, play a pivotal role in detecting latent vulnerabilities while assuring adherence to formally sanctioned risk management protocols [16]. Concurrently, the accelerating integration of technology into risk governance frameworks is noteworthy [14]. Deployment of sophisticated software platforms dedicated to risk oversight not only standardises workflows but also enhances measurement fidelity. Modalities such as EHR architectures, real‐time automated surveillance systems and advanced data analytics instruments collectively expedite risk identification, evaluation and attenuation. Equally, strategic alliances with external stakeholders merit attention [34]. Such collaborations broaden access to vetted practices, material assets and real‐time intelligence, thereby fortifying collective resilience in crisis scenarios and in the management of intricate risk profiles. Cultivating an organisation‐wide safety ethos emerges as a non‐negotiable enabler of enduring risk capability. A pervasive safety mindset compels personnel to embed risk oversight into habitual conduct. Embedding this ethos is realised through unwavering leadership endorsement, transparent channels of exchange, reinforcement of risk reporting without punitive sanction and decisive, nonretributive risk mitigation. These interdependent practices cultivate an environment in which safety becomes a shared operational imperative. Effective risk management in public health facilities, therefore, is not ancillary but central to sustained safety, enhanced clinical effectiveness and optimal resource stewardship. Comprehensive risk assessment frameworks must be systematically adapted to encompass the full spectrum of operational domains within the facility.

The purposeful integration of advanced technologies—specifically, EHRs, incident reporting systems and predictive analytics—provides a robust platform for early and accurate risk identification and management. Equally paramount is cultivating a safety and accountability culture among healthcare personnel. Such a culture is characterised by transparency, open dialogue and an unwavering commitment to lifelong learning, thereby empowering staff to proactively recognise and mitigate hazards. Interdisciplinary collaboration is an indispensable lever in this enterprise; the active participation of varied stakeholders in risk assessment and remedial planning produces strategies that are more inclusive and empirically sound. Systematic education and tailored training schemes systematically elevate personnel proficiency in risk management, ensuring that analytical knowledge is translated into operative skill. Ongoing assessment and iterative refinement of risk‐management systems are imperative to maintaining enduring efficacy. Consequently, facilities are obliged to routinely reassess governing policies, operational protocols and clinical outcomes, in conjunction with the subjection of performance metrics, internal audits and stakeholder perceptions to rigorous analytics. Such a comprehensive review regimen enables the establishment of performance baselines, the identification of emerging trends and the expeditious implementation of necessary corrective measures.

#### 2.5.3. Challenges of Implementing Risk Management Practices in Public Health Facilities

Establishing robust risk management protocols in finished public health facilities faces multifaceted barriers that, if unresolved, jeopardise both operational safety and clinical effectiveness. Foremost among these barriers is the entrenched resistance to procedural change among clinical and administrative personnel. Staff frequently perceive the introduction of risk mitigation frameworks as an encroachment on workflow rather than an aid, apprehensive that compliance will amplify both cognitive and physical burdens without correspondingly increased reward. Parallel constraints arise from the systemic scarcity of fiscal resources, endemic to the public health sector. Existing budgetary frameworks, already fragmented by competing operational imperatives, afford marginal latitude for reallocating funds to risk identification and management initiatives. In the absence of sustained investment, facilities are forced to operate with insufficient human capital, antiquated technological systems and training that occurs only by chance rather than by design. Such inadequacies, as noted by Phoya [11], expose clinical and administrative environments to treatable hazards that manifest when systemic defences are impaired. Further compounding these constraints, Whittaker et al. [35] empirically demonstrate that adherence to multiple regulatory and accreditation frameworks consumes disproportionate institutional bandwidth. Facilities remain perpetually engaged in collecting, analysing and reporting documentation, submitting to periodic third‐party assessments that, although vital for formal validation, episodically displace focus from organic and preventive risk management cycles.

Complementarily, Prakash and Ambekar [36] isolate cultural and organisational dynamics as systemic obstacles to risk management. Organisational cultures characterised by strong hierarchies, pronounced autonomy or persistent adherence to tradition often exhibit reluctance to embrace collaboration‐driven risk management strategies. Albasyouni et al. [37] further assert that technological impediments exacerbate the successful adoption of risk management frameworks. Within public health institutions, the challenge lies primarily in orchestrating the interoperability of heterogeneous technological platforms, including EHRs, risk assessment applications and incident‐reporting systems. The persistence of risk management activities is similarly jeopardised by the regular turnover of clinical and administrative personnel, compounded by fluctuations in leadership transitions. Newly appointed executives are positioned to modify, suspend or completely stigmatise existing risk management agendas, which, in tandem with divergent personal risk philosophies or educational backgrounds, frequently engenders policy fragmentation and uneven resource diversion.

### 2.6. Risk Assessment Tools and Methods for Building Projects

Isolated from casual defaults, organisations are compelled to institutionalise risk assessment instruments and methodological frameworks capable of delivering prospective identification, systematic analysis and risk appraisal [38]. Such devices vary in operational complexity, with variants that are predominantly quantitative or predominantly qualitative. Among the collection of established alternatives, organisations may encounter risk matrices, decision tree analysis and failure modes and effects analysis (FMEA), the latter being particularly popular across multidisciplinary applications.

Qualitative analyses of risk draw upon spontaneous observations, expert judgements and contextual narratives of specialists, whereas quantitative assessments rely on formal mathematical modelling and numerical estimates of probable or probable‐scoped setbacks or hazards [2]. The semi‐quantitative method reconciles the previous modalities by substituting explicit, formulaic synthesis of qualitative rankings and scaled metrics to calculate a single risk indicator. Existing applications of this assessment architecture may be divided according to the primary object of examination; the logic employed is as follows: Asset‐driven inquiry interrogates the comprehensive value of critical organisational components, threat‐focused inquiry interprets the nature and frequency of dangerous actors or events, and vulnerability‐oriented inquiry enumerates and assesses the exploitable weaknesses of the enterprise [39, 40].

Assessing risk during the planning and execution phases of construction projects typically employs a suite of methodologies [41]—risk matrices, FMEA and SWOT (strengths, weaknesses, opportunities, threats) analysis—each contributing distinct, complementary insights. Initial engagement with risk matrices enables project teams to classify identified hazards along two dimensions: probability of occurrence and severity of impact. The intersection of these dimensions enables practitioners to direct mitigating resources preferentially toward risks classified as both high‐likelihood and high‐impact. In contrast, the FMEA approach requires systematic decomposition of construction systems—be they processes, materials or assemblies—into hierarchically lower components or activities, followed by a rigorous inquiry into each component′s possible failure modes, their causal pathways, consequential effects and feasible preventive measures. SWOT analysis, by examining both endogenous and exogenous domains, functions as an overarching diagnostic: It explicitly maps a project′s salient capabilities and vulnerabilities (strengths and weaknesses) against the shifting commercial and environmental landscapes (opportunities and threats) [40, 42].

Among the arsenal of instruments available for systematic risk management in construction projects, decision trees, bow‐tie diagrams, project management software, structured risk assessment workshops and quantitative risk analyses merit particular consideration. The selection of an appropriate methodology is contingent upon an array of contextual variables, including project typology, magnitude and types of risk exposures, risk treatment strategies, regulatory compliance mandates, availability and reliability of primary and secondary data, collective judgement of domain experts and the quality of peer review processes. When strategic combinations of these instruments are judiciously implemented, it becomes feasible to formulate construction plans characterised by progressively diminished risk exposure [2, 39].

### 2.7. Research Gap

Although research on risk assessment and management practices (RAMP) has been extensively conducted in various countries, many of these studies have primarily concentrated on identifying critical success factors (CSFs) for building projects [43] and evaluating overall project success. Moreover, these studies have predominantly centred on developed countries, with limited attention to research in developing countries, particularly in Asia. Despite the existing literature on risk management practices in healthcare settings, a notable research gap remains in assessing the effect of risks on the performance of public health building projects in Dar es Salaam. Many studies focus on developed healthcare systems, and research is needed to examine risk management practices in developing countries like Tanzania. Factors such as unique regulatory environments, resource constraints and cultural considerations may significantly impact the implementation and outcomes of risk management strategies in these contexts.

### 2.8. Conceptual Framework

**Figure 1:**
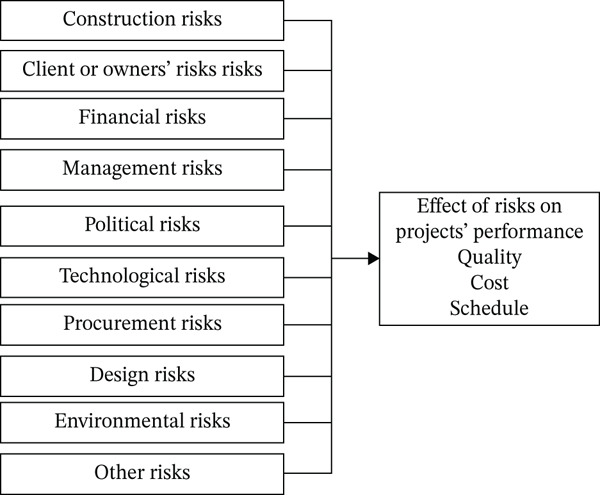
Conceptual framework.

The following framework depicts the relationship between the study variables; Figure 1 illustrates the connection between the variables, showcasing the presumed cause‐and‐effect relationship between the independent and dependent variables. The research framework proposes that the impact initiates with the fundamental components of risk management represented by independent variables such as risk identification, risk assessment, risk response and risk monitoring. It subsequently leads to the achievement of public health facilities as the study′s dependent variable.

## 3. Research Methodology

### 3.1. Research Approach

Generally, there are three approaches or methods for conducting research: qualitative methods, quantitative methods and mixed methods [44]. This study employed a quantitative research approach, with a comprehensive immersion in the local context, to assess the effects of risks encountered in public health building projects in Dar es Salaam. A quantitative research design is pertinent to make predictions, discover facts and test existing hypotheses [45]. The study was conducted in the Dar es Salaam region, focusing on public health building projects completed and ongoing in the municipal councils of Kinondoni, Ilala, Temeke, Ubungo and Kigamboni from 2017/2018 to 2023/2024. The choice of Dar es Salaam is reasonable because there are many public health projects. The unit of analysis was the public health building projects in Dar es Salaam from 2017/2018 to 2023/2024.

### 3.2. Sample Size Computation

In research, recruiting the entire population of interest is often neither appropriate nor feasible. Therefore, researchers typically recruit a sample from the population of interest for their study [46]. The sample is a subset of the total population. From this sample, insights and conclusions can be drawn about the entire population [47]. The study uses Yamane′s formula to determine the number of public health facilities in each municipality in Dar es Salaam. Equation (1) computes the sample size.

(1) n=N1+Ne2,

where ***n*** is the sample size to be determined, ***N*** is the selected population, and **e** is the level of precision or error sampling, normally expressed in percentages from ±5% to ±10% [48, 49]. However, in the case of this study, the level of precision was 10%. Given that the precision or sampling error level is ±10%. The study selected 56 public health projects from five municipalities in Dar es Salaam. The distribution of the sample of public health facilities in each municipality is presented in Table 1.

**Table 1:** Distribution of the sample of public health building projects in municipal.

Municipal	Number of health projects	Sample of health projects	
		Completed	Ongoing
Kinondoni	11	2	3
Ilala	32	7	8
Temeke	25	5	6
Ubungo	35	8	8
Kigamboni	21	6	4
**Subtotal**	**124**	**28**	**28**
**Total**		**56**	

### 3.3. Data Collection Methods

The systematic process of acquiring and measuring information on variables of interest is known as data collection. This process allows researchers to evaluate outcomes and address stated research questions. The study directly gathered primary data from the health building projects by administering a closed‐ended questionnaire. A questionnaire is a research instrument comprising a series of questions and prompts designed to collect information from respondents [50]. In the quantitative method, this study utilised closed‐ended questions for data collection. The study used a structured questionnaire (Appendix 1) to collect quantitative data on types of risks, risk management practices, challenges faced and project performance. The questionnaires were distributed to all public health building projects. The closed‐ended questionnaire was developed by adapting questions from previous studies, including those by Osipova [51] and [29]). Questionnaires must be carefully designed to ensure clarity and accurate interpretation of results.

### 3.4. Data Analysis

The study employs both descriptive and inferential data analysis techniques to generate findings and draw conclusions. All three research questions involved descriptive analysis. Such analysis included calculating the mean, standard deviation, relative importance index (RII) and normalising the mean scores.

#### 3.4.1. Risk Identification Process

**Figure 2:**
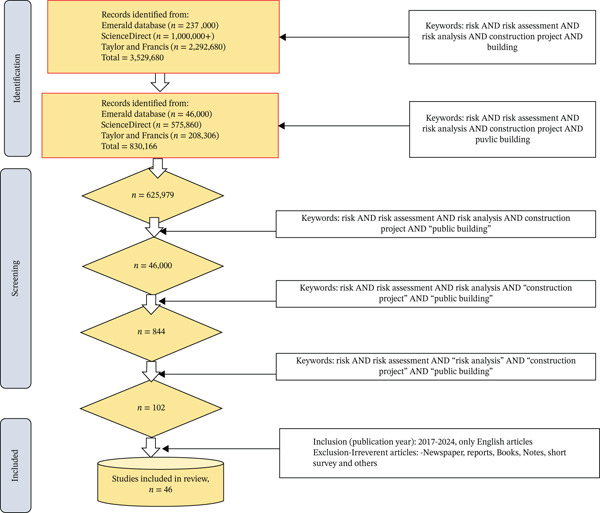
Article screening processes to identify risks.

All risks encountered in public health building projects were identified through a systematic literature review (SLR). The published articles were obtained from Emerald, ScienceDirect and Taylor & Francis databases. Only articles published between 2017 and 2024 were included in the risk identification process. This review excluded newspapers, books, duplicates, brief stories, reports, lecture notes, non–full articles, short surveys, articles not written in English and medical articles, as recommended by other SLR studies [52, 53]. In addition, the review did not take into account nonrelevant articles, that is, journal articles that did not mention risk, risk assessment, risk analysis, construction project or public building in either the abstract or the main text. Figure 2 illustrates the screening process from article identification to the final included articles.

#### 3.4.2. Risk Assessment

The first research question involved determining risks encountered in completed and ongoing public health building projects in Dar es Salaam. Three stages were considered to assess the risks and ultimately identify the risk level.

a. Risk identification: Potential risks were mentioned by a group of experts selected from five building projects. Those members had at least 5 years of experience in building projects. They identified risks and categorised them into financial risks, management risks, political risks, technological risks, procurement risks, design risks, environmental risks, construction risks, client or owner risks and other risks. The categories of risks were mainly extracted from previous studies, including studies by Martin et al. [54], Pascarella et al. [55] and [29].
b. Risk analysis: The determination of risk is mostly referred to as risk analysis. This process comprised the estimation of severity (risk impact) and risk likelihood (risk occurrence). The rating scale guided the risk analysis, as shown in Table 2. Equation (2) was used to compute the risk scores.

(2) Risk=Likelihood×Severity impact

c. Likelihood determination: The likelihood of each risk for the buildings was determined by categorising the risks into five:
  i. Low (1): Nearly impossible to happen during the project execution; no immediate action was needed.
  ii. Low–medium (2): Moderately unlikely to happen during the project execution; minimal action was needed.
  iii. Medium (3): Likely to occur; actions were taken to reduce or control the risk.
  iv. Medium–high (4): Management or organisers began to mitigate more than likely to occur.
  v. High (5): High probability that the risk occurred; immediate action plans required.

**Table 2:** Risk assessment scoring tool.

Impact/effect/severity	Likelihood
(1) **Insignificant:** Insignificant impact on health building projects	(1) **Low:** Nearly impossible to happen during the project execution, and no immediate action was needed
(2) **Mild:** Minimal impact on health building projects	(2) **Low–medium:** Moderately unlikely to have happened during the project execution, and minimal action was needed
(3) **Moderate:** Could delay operations in completing the project, affect short‐term programs, require moderate management effort, and may draw publicity	(3) **Medium:** Likely occurred, and actions were taken to reduce or control the risk
(4) **Significant:** A significant effect on long‐term programs could result in major project failure in terms of performance	(4) **Medium–high:** More than likely to occur, and management or organisers began to mitigate
(5) **Catastrophic:** Long‐term and serious effect on health building projects	(5) **High:** High probability the risk occurred; immediate action plans required

Then, a group of experts selected from five building projects ranked the likelihood of all risks. Those members had at least 5 years of experience in building projects. Expert review panels typically involve three to six experts for risk rating in research, with five being the ideal number for balancing quality and efficiency [56, 57].

**Figure 3:**
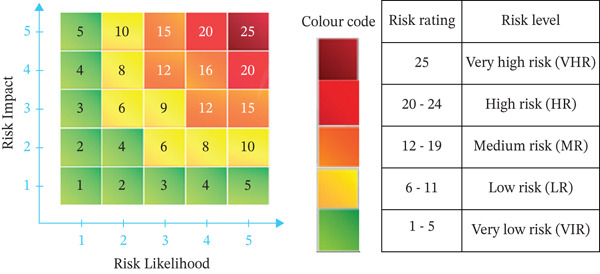
Risk ratings and risk levels. *Source:* Modified from Pascarella et al. [55] and Proto et al. [60].

The risk categories were mainly derived from previous studies, including those by Gama et al. [58] and Farooqui and Tauheed [59].

d. Risk rating: The rating process adopted the ranking of risks as outlined by Pascarella et al. [55] and Proto et al. [60]. Figure 3 highlights the risk ratings and risk levels.

#### 3.4.3. Regression Analysis

The study uses regression analysis as an inferential data analysis method. The regression method enables the study to assess the impact of independent variables on dependent variables, as specified by the research questions. The study quantifies the influence of various factors (such as technological risks, financial risks, design risks, client or owner risks, management risks, construction risks, political risks, environmental risks, procurement risks and other risks) on the outcome of the overall building project performance and then develops a model for each. Equation (3) represents the regression model, indicating the dependent variable (*Y*_1_) against the independent variables (*X*_1_ to *X*_10_).

(3) Y1=β0+β1X1+β2X2+β3X3+β4X4+β5X5+β6X6+β7X7+β8X8+β9X9+β10X10+ε,

where *X*_1_–*X*_10_ = technological, financial, design, client or owner, management, construction, political, environmental, procurement and other risks, respectively.

In assessing the risks associated with ongoing projects, regression analysis was used as one of the tools that included ANOVA to analyse risks [42]. This is because ANOVA and regression models can execute the analysis by comparing means across multiple risks encountered, determining significant differences and relationships among variables and evaluating risks in line with construction and operational risk [61]. Likewise, ANOVA can help understand the variability of encountered risks by providing information on variability levels within a regression model [62].

#### 3.4.4. Reliability Test

Project data should be tested to establish validity. So, all data from 56 health building projects were tested to measure the internal consistency of the responses. SPSS 21.0 tested reliability using Cronbach′s alpha, as suggested by studies such as Hodson [63] and Amora [64]. The most acceptable internal consistency should be when Cronbach′s alpha is at least 0.7 [49, 65, 66].

#### 3.4.5. Criticality Analysis of Risk Management Practices

Appendix 1 comprises a list of risk management practices. The subcomponents for each practice were identified from various literature sources, including Ahmed [67], MOF [68] and Shrivastava et al. [69]. This study considered risk management practices. Those practices were analysed by establishing the criticality via SPSS 21.0. Then, the final values for the criticality were obtained by normalising the mean scores as per Hammad et al. [70] and Abu Dabous et al. [39]. The normalisation values (NVs) were calculated using Equation (4):

(4) NVs=Mean scores−Minimum meanMaximum mean−Minimum mean.

#### 3.4.6. Relative Important Index

After the normalisation of the mean scores in Section 3.4.6, the RII was also calculated. Equation (5) was used to calculate the RII, which ranges from 0 to 1, with the highest RII. The RII of values is categorised into five levels: low (L) (0 ≤ *RI*<0.2), medium–low (M‐L) (0.2 ≤ *RI* < 0.4), medium (M) (0.4 ≤ RI < 0.6), high–medium (H‐M) (0.6 ≤ RI<0.8) and high (H) (0.8 ≤ *RI* ≤ 1) [71, 72].

(5) RII=ΣWΑ×Ν,

where *W* = the weight score of each risk management practice, *A* = the highest rank on the scale (5 in this study) and *N* = the total number of respondents.

### 3.5. Ethical Consideration

The study was conducted in the Dar es Salaam region by considering the public health building projects that have been completed and ongoing in Kinondoni, Ilala, Temeke, Ubungo and Kigamboni Municipal Councils from 2017/2018 to 2023/2024. Thus, some individuals were involved in the study. Therefore, before collecting data, the ethical issues were first cleared. All teams for the public health building projects were well informed about transparency and accountability, voluntary participation, respect for participants, informed consent, confidentiality and anonymity. The Mechanical and Industrial Engineering Department approved the data collection clearance on behalf of the university′s ethical committee. The approval had the reference number 2018‐06‐00494. Participants were provided with the informed consent forms, and the study was conducted only with those who had read and understood the informed consent sent or provided to them.

## 4. Results and Discussions

### 4.1. Reliability Test

Data from 56 health building projects were analysed to assess the internal consistency of responses. SPSS 21.0 tested the reliability by establishing Cronbach′s alpha values, as shown in Table 3. According to Stentoft et al. [73], the most acceptable internal consistency should be when Cronbach′s alpha is at least 0.7. The overall Cronbach′s alpha for the collected data was 0.809, indicating that the data were reliable.

**Table 3:** Reliability results for the collected data on health building projects.

Item category	Measured items	Cronbach′s alpha	Cronbach′s alpha based on standardised items	No. of items
Risks	Financial risks	0.862	0.921	7
	Management risks	0.806	0.909	5
	Political risks	0.913	0.971	4
	Technological risks	0.85	0.812	3
	Procurement risks	0.859	0.756	6
	Design risks	0.921	0.863	6
	Environmental risks	0.856	0.806	3
	Construction risks	0.859	0.809	7
	Client or owners′ risks	0.921	0.871	5
	Other risks	0.872	0.848	5

Performance of public health building projects	Cost, Schedule and Quality	0.879	0.849	3

Risk management practices	Avoid or eliminate	0.901	0.871	5
	Mitigation	0.87	0.892	11
	Transfer	0.879	0.849	2
	Acceptance	0.941	0.911	4

**Overall**			**0.809**	**76**

### 4.2. Assessment of Risk Levels

#### 4.2.1. Risk Identification Process

**Figure 4:**
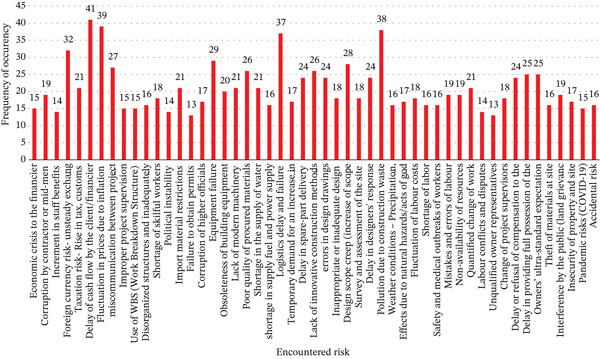
A total of encountered risks in the public building construction project.

The risk identification process was performed using the SLR (Figure 2). A total of 46 articles extracted from ScienceDirect, Emerald and Taylor & Francis resulted in an analysis of all key risks published for the building construction projects. Figure 4 depicts the histogram for all encountered risks. The most reported risks include the delay of the cash flow by the client (41), price fluctuation (39), pollution due to construction waste (38) and logistics delay and failure (37). The least occurred risks are failure to obtain permits and an unqualified owner′s representative, both of which occurred 13 times.

#### 4.2.2. Risk Analysis for Public Health Projects

Following the collected data from 56 health building projects, the study by Pascarella et al. [55] and Proto et al. [60] guided the analysis from risk identification to risk rating. Consequently, the procedures in Section 3 were followed to identify the most critical risks. Table 4 depicts all risks which were identified and ranked for the 56 building projects. Figure 3 shows that when the risk score is 25, that means the risk level is very high risk, high risk (20–24), medium risk (12–19), low risk (6–11) and very low risk (1–5). Therefore, the analysis in Table 4 shows that 56 projects had no high or very high risks. However, the majority of the risks identified and ranked are at very‐low‐risk levels because their risk scores were between 1 and 5. The medium level had five risks, including the delay of cash flow by the contractor, fluctuation in prices due to inflation and pollution due to construction waste. Such risks align with the risks identified by other researchers, including Gama et al. [58] and Pascarella et al. [55].

**Table 4:** Risks encountered in the health building projects.

Risk category		Risk	Impact	Likelihood	Risk score	Risk level
Financial risks	FR1	Economic crisis for the financier	2.23	1.21	2.70	Very low
	FR2	Corruption by contractor or middlemen	1.52	3.03	4.61	Very low
	FR3	Increment in staff benefits	1.68	1.09	1.83	Very low
	FR4	Foreign currency risk—unsteady exchange rates	3.59	3.11	11.16	Low
	FR5	Taxation risk—rise in tax and customs	3.21	1.72	5.52	Very low
	FR6	Delay of cash flow by the contractor	4.2	3.71	15.58	Medium
	FR7	Fluctuation in prices due to inflation	4.04	3.55	14.34	Medium

Management risks	MR1	Miscommunication between project stakeholders	2.75	3.16	8.69	Low
	MR2	Project supervision	2.54	1.05	2.67	Very low
	MR3	Use of WBS (work breakdown structure)	1.84	1.35	2.48	Very low
	MR4	Disorganised structures and inadequately qualified staff	2.63	1.14	3.00	Very low
	MR5	Shortage of skilled workers	3.98	1.01	4.02	Very low

Political risks	PR1	Political instability	1.71	1.22	2.09	Very low
	PR2	Import material restrictions	3.23	1.74	5.62	Very low
	PR3	Failure to obtain permits	1.57	1.08	1.70	Very low
	PR4	Corruption of higher officials	1.57	2.08	3.27	Very low

Technological risks	TR1	Equipment failure	3.93	2.44	9.59	Low
	TR2	Obsoleteness of building equipment	3.21	1.52	4.88	Very low
	TR3	Lack of modern machinery	3.86	1.37	5.29	Very low

Procurement risks	RP1	Poor quality of procured materials	3.68	2.19	8.06	Low
	RP2	Shortage in the supply of water	3.21	1.72	5.52	Very low
	RP3	Shortage in the supply of fuel and power supply	2.2	1.31	2.88	Very low
	RP4	Logistics delay and failure	3.93	3.44	13.52	Medium
	RP5	Temporary demand for an increase in materials	2.8	1.31	3.67	Very low
	RP6	Delay in spare part delivery	3.93	1.84	7.23	Low

Design risks	DR1	Lack of innovative construction methods required	3.71	2.22	8.24	Low
	DR2	Errors in design drawings	3.45	1.96	6.76	Low
	DR3	Inappropriate or inadequate design	3.68	1.09	4.01	Very low
	DR4	Design scope creep (increase of scope over time)	3.86	2.37	9.15	Low
	DR5	Survey and assessment of the site	2.41	1.72	4.15	Very low
	DR6	Delay in the designers′ response	3.36	2.09	7.02	Low

Environmental risks	ER1	Pollution due to construction waste	3.27	4.27	13.96	Medium
	ER2	Weather conditions—precipitation, temperature and humidity	2.68	1.19	3.19	Very low
	ER3	Effects due to natural hazards/acts of God—floods, earthquakes etc.	2.86	1.27	3.63	Very low

Construction risks	CR1	Fluctuation of labour costs	2.91	1.42	4.13	Very low
	CR2	Shortage of labour	3	1.05	3.15	Very low
	CR3	Safety and medical outbreaks among workers	2.02	1.53	3.09	Very low
	CR4	Mistakes and errors of labour	2.95	1.46	4.31	Very low
	CR5	Non‐availability of resources	3.23	1.34	4.33	Very low
	CR6	Quantified change of work	2.23	2.55	5.69	Very low
	CR7	Labour conflicts and disputes	2.04	1.07	2.18	Very low

Client or owner risks	OR1	Unqualified owner representatives	1.02	1.53	1.56	Very low
	OR2	Change of project supervisors	3.96	1.01	4.00	Very low
	OR3	Delay or refusal of compensation to the contractor	4.14	1.65	6.83	Low
	OR4	Delay in providing full possession of the site	3.79	1.93	7.31	Low
	OR5	Owners′ ultrastandard expectation	1.75	4.26	7.46	Low

Other risks	RO1	Theft of materials at the site	2.09	1.49	3.11	Very low
	RO2	Interference by the public (land grievances, strikes etc.)	2.36	1.87	4.41	Very low
	RO3	Security of property and site	2.29	1.48	3.39	Very low
	RO4	Pandemic risks (COVID‐19)	2.38	1.12	2.67	Very low
	RO5	Accidental risk	2.54	1.25	3.18	Very low

#### 4.2.3. Risk Visualisation

**Figure 5:**
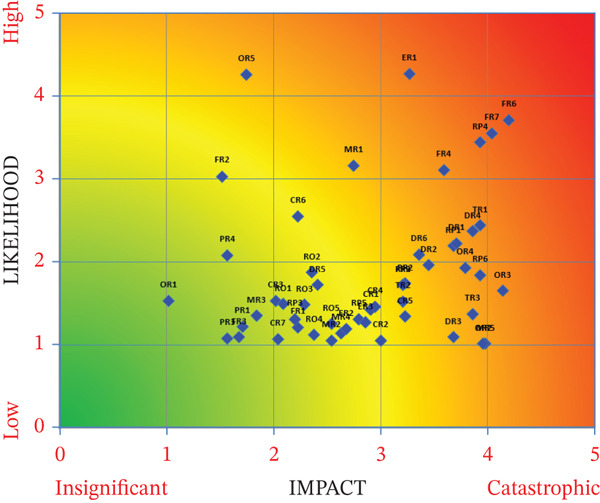
Risk heat map presenting the encountered risks.

Following the analysis of all identified risks across 56 public building projects, a risk map (risk heat map) was used to visualise them. A risk heat map as a data visualisation tool was deployed to present risks for specific health building projects. Figure 5 depicts the risk heat map as drawn by Microsoft Excel 2021. The figure comprises likelihood and impact (severity). The likelihood ranges from low to high, whereas the impact or severity ranges from insignificant to catastrophic (Table 2). Equation (2) computes the risk scores, whereas Figure 3 was used to rank the risk scores using specific colour codes as adapted from Pascarella et al. [55] and Proto et al. [60].

Therefore, the performed risk analysis depicted in Table 4 and Figure 5 signifies that all engaged building projects had low‐to‐medium‐level risks. All stakeholders, including the government, users and others, should be well notified by looking at the magnitude of a risk. Attention should be paid to these risks to avoid the risk increase. Despite the risks being at low‐to‐medium levels, the impact (severity) and likelihood can increase in the future, depending on the circumstances and operational environments of the building projects. The findings align with those of Pascarella et al. [55] and Proto et al. [60]. Pascarella et al. [55] also highlighted that people involved in projects should take control by understanding the consequences that could result if the identified threat occurs.

### 4.3. Linearity Assessment Test

**Figure 6:**
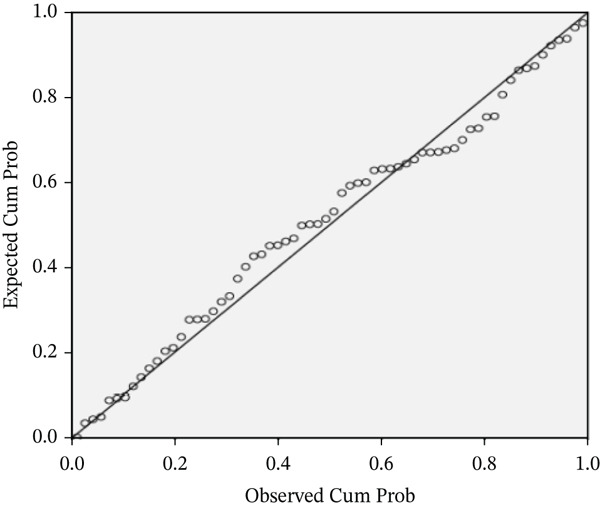
Normal P–P plot of residuals.

SPSS 21.0 was used to conduct the linearity test to determine the linear relationship between the predictor and dependent variables. A linear relationship is presumed to be a prerequisite for linear regression [74]. The use of a nonlinear regression model would be necessitated by nonlinearly related variables. The linear relationship between predictors and the outcome variable was confirmed by the linearity test results, as evidenced by the normal P–P plot (Figure 6), which confirmed that the points fell along the linear equation. The more linear the relationship between predictors and the outcome variable, the closer the residuals are to the straight line [75].

### 4.4. Regression Analysis to Explore the Effects

SPSS 21.0 software was used to conduct regression analysis; regression analysis usually provides three tables, which are used to make decisions regarding the model. The analysis of variance (ANOVA) table shows how well the model predicts the data, the regression results table displays the impact of each variable on the dependent variable, and the model summary displays how well the model predicts the data. The *R* ^2^ value (0.727) in Table 5 illustrates that 72.7% of the variations in the dependent variable could be explained by the model′s independent variables [76]. It further shows that 27.3% of the variations in the performance of public health building projects are accounted for by other factors not accounted for in this model. The Durbin Watson values range from 0 to 4; the Durbin Watson value of 2 indicates no autocorrelation. The value for this model was 2.152, which indicated the absence of autocorrelation between variables [77].

**Table 5:** Regression model summary.

Model	*R*	*R* ^2^	Adjusted *R* ^2^	Std. error of the estimate	Durbin–Watson
1	0.853^a^	0.727	0.693	0.53017	2.152

^a^Predictors: (constant), technological risks, financial risks, design risks, client or owner risks, management risks, construction risks, political risks, environmental risks, procurement risks and other risks.

The ANOVA results in Table 6 show the model significance; the *p* value of the model was < 0.001, which is less than 0.05, suggesting that the model was a significant predictor of the outcomes with a significant influence between at least one independent variable and the dependent variable [78]. Consequently, the results were interpreted by analysing the individual regression coefficients (beta values and associated *p* values) for each predictor variable in the model. Post hoc tests or simple effects analyses were not performed, as they are generally more relevant in experimental designs or when comparing group means in categorical ANOVA contexts [79, 80]. This study employed multiple linear regression to estimate the individual contributions and statistical significance of each predictor variable while controlling for others. Consequently, the subsequent analysis concentrated on the importance and extent of each risk factor coefficient to elucidate their particular impacts on project performance.

**Table 6:** The ANOVA test results for regression analysis.

Model		Sum of squares	Degree of freedom (df)	Mean square	*F*	Sig.
1	Regression	37.600	10	3.76	10.899	< 0.001^b^
	Residual	15.515	45	0.345		
	Total	53.115	55			

^b^Predictors: (constant), technological risks, financial risks, design risks, client or owner risks, management risks, construction risks, political risks, environmental risks, procurement risks and other risks.

The regression coefficients in Table 7, which are also incorporated in Equation (6), explain the effect of each risk category on the overall performance of public health building projects. The coefficient for the constant term (3.460) explains changes in the performance of public health building projects captured by this model, without considering any of the independent variables.

(6) Y1=3.4600.6850.3040.2570.2910.1520.6060.3740.3230.4670.055−X1−X2−X3−X4−X5−X6−X7−X8−X9−X10+ε,

**Table 7:** Regression model coefficients.

Model		Unstandardised coefficients		Standardised coefficients	t	Sig.
		B	Std. Error	Beta		
1	(Constant)	3.460	1.706		2.028	0.048
	Financial risks	‐0.685	0.118	‐0.136	‐5.806	<0.001
	Management risks	‐0.304	0.083	‐0.109	‐3.677	0.012
	Political risks	‐0.257	0.146	‐0.365	‐1.767	0.084
	Technological risks	‐0.291	0.123	0.335	‐2.366	0.041
	Procurement risks	‐0.152	0.076	‐0.141	‐1.981	0.047
	Design risks	‐0.606	0.203	‐0.074	‐2.983	0.031
	Environmental risks	‐0.374	0.197	0.064	‐1.903	0.049
	Construction risks	‐0.323	0.106	0.017	‐3.047	0.024
	Client or owner risks	‐0.467	0.115	‐0.207	‐4.061	<0.001
	Other risks	‐0.055	0.229	‐0.038	‐0.238	0.813

where *Y* = project performance and *X*_1_–*X*_10_ = financial risks, management risks, political risks, technological risks, procurement risks, design risks, environmental risks, construction risks, client or owner risks and other risks, respectively.

#### 4.4.1. Effect of Financial Risks on the Performance of Public Health‐Buildings

Financial risks with a beta coefficient (−0.685) had the strongest effect on the performance of public health building projects, and a *β* value of −0.685 indicated that a unit increase in financial risks reduces the performance of public building projects by 0.685 units. The result of the significance test in Table 7 shows that the effect of financial risks at a 95% confidence level was significant with a *p* value of < 0.001 [81]. The financial risks factor (*β* = −0.685, *p* < 0.001) pertains to the fiscal and macroeconomic context of Tanzania that pertains to the government and donor trust funding. It becomes evident that public health infrastructure projects are financially limited. There are financial challenges caused by project funding interruptions, variable exchange rates for construction materials and inflationary pressures on construction costs. These challenges explain the dominance of financial risks and the gaps in the system.

#### 4.4.2. Effect of Management Risks on the Performance of Public Health‐Buildings

The study revealed a negative effect of management risks on the overall performance of public health building projects, with risks such as project supervision and communication among stakeholders included in the management risks; the analysis showed that a unit increase in management risks leads to a decrease in the performance of public health building projects by 0.304. Also, this study found that the effect was significant at the 95% confidence level (*p* = 0.012). The particular influence of management risks (*β* = −0.304, *p* = 0.012) and client/owner risks (*β* = −0.467, *p* < 0.001) pertains to the social aspect of management, in particular, the fragmentation of project goals and the bureaucracies and gaps in the management chain in the public construction projects. These report challenges in the vertical and horizontal controls at the management level, which in turn affect the outcomes of the projects.

#### 4.4.3. Effect of Political Risks on the Performance of Public Health‐Buildings

Political risks had a beta value of −0.257 in Table 7, which explains that political risks had a negative impact on the overall performance of public health building projects. A unit increase in political risks would decrease project performance by 0.257 units. However, the influence of political risks, which included political instability and restrictions on importation, on the performance of public health building projects was not significant at the level of significance chosen for this study, that is, *α* = 0.05, to which the political risk variable had a *p* value of 0.084, which is higher than 0.05.

#### 4.4.4. Effect of Technological Risks on the Performance of Public Health‐Buildings

The regression analysis yielded a beta coefficient of −0.291, indicating that the performance of public health building projects will decrease by 0.291 units for each unit increase in technological risks. This effect was further proved to be significant with the help of *p* values (Sig.) at the *α* = 0.05 level of significance, as it had a *p* = 0.041, which was below 0.05. The impact of technological risks (*β* = −0.291, *p* = 0.041) is due to the level of technology and the construction resources for project execution. Although the risk of losses due to changes in the political environment are not statistically significant (*β* = −0.257, *p* = 0.084), they do reflect the lack of a defined regulatory framework that controls and provides a strong mandate for the changes in the management scope of the system in terms of the changes that affect the projects.

#### 4.4.5. Effect of Procurement Risks on the Performance of Public Health‐Buildings

A beta coefficient of −0.152 for procurement risks showed the second least effect on public health building project performance, as a unit increase in political risks decreased project performance by 0.152 units. The outcome of the significance test displayed in Table 7 indicates that the effect of political risks was statistically significant at a 95% confidence level, as evidenced by a *p* value of 0.047. The impact of procurement risks (*β* = −0.152, *p* = 0.047) signals challenges in public procurement, including compliance delays, procurement readiness and limited supplier capabilities. These factors all affect the procurement of construction materials and services.

#### 4.4.6. Effect of Design Risks on the Performance of Public Health‐Buildings

The regression results found that design risks, including design scope creep and errors, had a detrimental impact on the overall performance of public health building projects. The analysis indicated that a 0.606 decrease in performance was associated with each unit increase in design risks. Additionally, the regression results discovered that this effect was deemed statistically significant with a *p* value of 0.031 at a confidence level of 95%.

#### 4.4.7. Effect of Environmental Risks on the Performance of Public Health‐Buildings

The beta coefficient for environmental risks in the regression analysis was −0.374, indicating that for every unit increase in technological risks, the performance of public health building projects will decrease by 0.374 units. The significance of this effect was further supported by *p* values (Sig.) at the *α* = 0.05 level, with a *p* = 0.049, just below the threshold of 0.05.

#### 4.4.8. Effect of Construction Risks on the Performance of Public Health‐Buildings

The regression findings showed that construction risks such as labour errors, safety issues and worker health outbreaks negatively affected the overall performance of public health building projects. The study showed that for every increase in construction risks, there was a corresponding decrease in performance of 0.323 units. Moreover, the regression analysis found that this effect was statistically significant, with a *p* value of 0.024 at a 95% confidence level.

#### 4.4.9. Effect of Client or Owner Risks on the Performance of Public Health‐Buildings

The regression analysis revealed that client/owner risks, such as delayed compensation, site‐handling delays and high expectations from the owner, negatively affect the performance of public health building projects. The study found that with each additional unit of design risk, there was a corresponding decrease of 0.467 in performance. Furthermore, the regression analysis revealed that this impact was statistically significant (*p* < 0.001 at the 95% confidence level).

#### 4.4.10. Effect of Other Risks on the Performance of Public Health‐Buildings

The beta value of −0.055 for the risk category labelled as other risks in Table 7 indicates that these risks had a negative effect on the overall effectiveness of public health building projects. An increase of one unit in these risks would result in a 0.055‐unit decline in project performance. Nonetheless, the impact of these risks, such as accidents and material theft, on the overall performance of public health building projects did not reach statistical significance in this study with a chosen level of *α* = 0.05. One risk variable had a *p* value of 0.084, exceeding the threshold of 0.05.

In summary, the regression model for the effect of the assessed risks on the performance of public health‐buildings showed that there was a negative effect on the performance for all the measured risks (technological risks, financial risks, design risks, client or owner risks, management risks, construction risks, political risks, environmental risks, procurement risks and other risks). However, for the significance of those effects, the analysis demonstrated greater significance (i.e., *p* < 0.001) for two categories of risk: financial risks and client/owners′ risks. Subsequently, there was a significant effect on the performance of public health building projects from the other six categories of risks (technological risks [*p =* 0.041], design risks [*p =* 0.031], management risks [*p =* 0.012], construction risks [*p =* 0.024], environmental risks [*p =* 0.049] and procurement risks [*p =* 0.047]). The effects and significance of the risk categories on each public building project performance indicator (cost, schedule and time) are presented in Table 8 through unstandardised beta coefficients and *p* values. The corresponding regression model equations for cost, schedule, and time are presented in Equations ((7)–(9)), respectively.

**Table 8:** Regression coefficients and significance of risks on performance indicators.

Model		Unstandardised coefficients			Sig. 1 (cost)	Sig. 2 (schedule)	Sig. 3 (quality)
		*B*_1_ (cost)	*B*_2_ (schedule)	*B*_3_ (quality)			
1	(Constant)	4.029	2.338	5.014	0.031	0.019	0.044
	Financial risks	−0.443	−0.216	−0.133	0.046	0.027	0.005
	Management risks	−0.045	−0.524	−0.007	0.906	0.046	0.017
	Political risks	−0.054	−0.407	−0.189	0.858	0.005	0.562
	Technological risks	−0.257	−0.378	−0.194	0.012	0.211	0.376
	Procurement risks	−0.168	−0.285	−0.104	0.019	0.494	0.732
	Design risks	−0.219	−0.629	−0.115	0.040	0.109	0.031
	Environmental risks	−0.078	−0.255	−0.123	0.766	0.509	0.660
	Construction risks	−0.353	−0.412	−0.331	0.024	0.353	0.005
	Client or owner risks	−0.459	−0.437	−0.353	0.043	0.046	0.099
	Other risks	−0.261	−0.277	−0.233	0.182	0.332	0.264

Regression equations are as follows:

(7) Cost=4.0290.4430.0450.0540.2570.1680.2190.0780.3530.4590.261−X1−X2−X3−X4−X5−X6−X7−X8−X9−X10+ε,

(8) Schedule=2.3380.2160.5240.4070.3780.2850.6290.2550.4120.4370.277−X1−X2−X3−X4−X5−X6−X7−X8−X9−X10+ε,

(9) Quality=5.0140.1330.0070.1890.1940.1040.1150.1230.3310.3530.233−X1−X2−X3−X4−X5−X6−X7−X8−X9−X10+ε,

where *Y* = project performance and *X*_1_–*X*_10_ = financial risks, management risks, political risks, technological risks, procurement risks, design risks, environmental risks, construction risks, client or owner risks and other risks, respectively.

Generally, the findings substantiate that the perceived risk–performance correlations cannot be merely considered as arbitrary statistical incidents; on the contrary, they are integrated outcomes of the regional economic, institutional and policy framework. This contextual complexity justifies the development of risk management mechanisms that are specifically directed to the structural and operational issues of the public health building projects in Tanzania.

### 4.5. Risk Management Practices for the Public Health Building Projects

After assessing risk levels and their effects on the performance of public health building projects, the study developed risk management practices to address risks in these projects. The risk management approaches assessed included avoidance, which suggests reducing the threat or risk; mitigation, which suggests eliminating the probability or the effect of the risk; transfer, which suggests shifting the responsibility and effect of the threat to a third party; and acceptance, which acknowledges the risk and tolerates it without executing immediate action. These categories had subcomponents which were assessed using a structured questionnaire.

The suggested risk management practices were analysed through the RII and normalisation of the mean scores. The RII helped to rank the management practices according to their importance, whereas the normalisation of the mean score helped to rank and understand which factors were critical, as utilised by studies [82, 83].

The findings of the RII in Table 9 reveal that two management practices, ARM1, removing the source of the threat, and ARM4, changing the project management plan in terms of personnel, were ranked highest with RII (0.71); either these two practices together with ARM5, change the project management plan in terms of technology, were found to be critical under the criticality assessment with normalisation values higher than 0.5 [82, 84]. For the case of the risk mitigation practices, MRM8, choosing different suppliers, and MRM9, outlining the requirements clearly, had the highest RII ranking of 0.81, whereas criticality analysis shows that all items were critical except for three items MRM1, employee or workforce training via workshops and seminars; MRM2, regular audits and updates; and MRM5, using fewer complex design and construction procedures were not critical with normalisation values less than 0.5. Only one item for each risk transfer and acceptance was not critical; such items were TRM2, outsourcing project activities that lack in‐house expertise, and ACM1, performing the opportunity cost (trade‐off) between the benefits and disbenefits of the risks. The risk management actions for each risk management practice are in Appendix 1.

**Table 9:** Risk management practices for public building projects.

Category	Code	Mean	Weight	RII	Normalisation	Criticality
Avoid or eliminate	ARM1	3.54	198.0	0.71	1.00	Critical
	ARM2	1.98	111.0	0.40	0.00	Not critical
	ARM3	2.21	124.0	0.44	0.15	Not critical
	ARM4	3.48	198.0	0.71	0.96	Critical
	ARM5	3.27	183.0	0.65	0.83	Critical

Mitigation	MRM1	3.23	181.0	0.65	0.25	Not critical
	MRM2	3.34	187.0	0.67	0.33	Not critical
	MRM3	4.14	232.0	0.83	0.91	Critical
	MRM4	4.27	239.0	0.85	1.00	Critical
	MRM5	2.88	161.0	0.58	0.00	Not critical
	MRM6	3.93	220.0	0.79	0.76	Critical
	MRM7	3.84	215.0	0.77	0.69	Critical
	MRM8	4.07	228.0	0.81	0.86	Critical
	MRM9	4.05	227.0	0.81	0.84	Critical
	MRM10	3.64	204.0	0.73	0.55	Critical
	MRM11	3.89	218.0	0.78	0.73	Critical

Transfer	TRM1	3.86	216.00	0.77	1.00	Critical
	TRM2	3.82	214.00	0.76	0.00	Not critical

Acceptance	ACM1	3.63	203.00	0.73	0.00	Not critical
	ACM2	4.04	226.00	0.81	0.89	Critical
	ACM3	3.89	218.00	0.78	0.57	Critical
	ACM4	4.09	229.00	0.82	1.00	Critical

## 5. Conclusion and Recommendations

### 5.1. Conclusion

Public health building projects encounter several risks throughout all the project phases. The occurrence of risks fosters the need to identify, analyse and present risks using a risk heat map and matrix score diagram. So, this study assessed risks encountered by the completed projects within the health building projects. The findings highlight the need to continue assessing potential risks encountered when executing public building projects. Through the quantitative research approach, the study has assessed risks encountered, assessed the effect or influence of the risks encountered by the public health building projects and proposed risk management practices that can enhance project performance. Fifty‐six projects were involved during the data collection. SPSS 21.0 and Microsoft Excel were used to analyse the collected data, including the regression analysis and linearity test. Microsoft Excel assisted in drawing the risk heat map. Before analysing the data, the reliability was tested, and Cronbach′s alpha values were determined for the risks, the risk influence and the risk management responses. The overall Cronbach′s alpha value for the data collected was 0.809, which indicates that the data were reliable.

The findings conclude that most risks were low, leaving fewer risks at the medium level. Examples of the risks ranked at the medium level include the delay of cash flow by the contractor, fluctuation in prices due to inflation and pollution due to construction waste. The risk heat map visualised all risks by indicating the severity (impact) and the likelihood (occurrence) of each identified risk.

Furthermore, the regression model for the effect of the assessed risks on the performance of public health‐buildings showed that there was a negative effect on the performance for all the measured risks (technological risks, financial risks, design risks, client or owner risks, management risks, construction risks, political risks, environmental risks, procurement risks and other risks). However, for the case of the significance of those effects, the analysis demonstrated a higher significance (i.e., *p* < 0.001) for two categories of risks: financial risks and client/owners′ risks. Subsequently, there was a significant effect on the performance of public health building projects from the other six categories of risks (technological risks [*p =* 0.041], design risks [*p =* 0.031], management risks [*p =* 0.012], construction risks [*p =* 0.024], environmental risks [*p =* 0.049] and procurement risks [*p =* 0.047]). The effect and significance of the risk categories on each of the public building project performance indicators (cost, schedule and time) are presented through unstandardised beta coefficients and *p* values. The study has successfully added to the board′s knowledge of project risk management and performance, especially in building projects. The risks encountered can be controlled using the proposed risk management practices. Implementing risk management practices could help the risk analysis to avoid negative impacts on project quality, scheduled time and budget. In conclusion, the study has demonstrated that, despite the many risks and risk factors analysed in public health projects being rated at low to medium levels, they still exert a statistically significant negative influence on project performance—particularly financial and client‐related risks—therefore necessitating continuous monitoring and proactive management. The adoption of structured and adaptive risk management practices is therefore essential to mitigate potential impacts and enhance project outcomes in terms of cost, time and quality.

### 5.2. Recommendations

A flexible, interdisciplinary and complementary approach to risk management in building initiatives is necessary to account for the constantly evolving nature of risk factors (qualitative and quantitative). In addition, it requires a detailed account and elucidation of the mechanisms used to execute initiatives. One recommendation is that various building projects implement the proposed risk management practices to avoid potential severe issues. The results indicate that all engaged building projects had low to medium risk. All stakeholders, including the government, users and others, should be well notified based on the magnitude of the risk. Attention should be paid to these risks to avoid risk increases. Despite the risks being at low to medium levels, the impact (severity) and likelihood can increase in the future, depending on the circumstances and operational environments of the building projects. Risk management practices for avoiding, escalating, transferring, accepting and mitigating risks are important for improving building project performance.

### 5.3. Significance of the Study

The study′s findings offer valuable insights into the strengths and weaknesses of current risk management practices in public health facilities. Such insights are pivotal in shaping and refining government policies concerning building projects, potentially leading to the development of more effective guidelines and regulations to ensure successful project outcomes. Effective risk management is crucial for safeguarding an organisation′s assets and resources from potential threats and uncertainties, including financial assets, intellectual property, human resources and infrastructure. A robust risk management framework upholds a positive reputation, fostering trust and engagement among stakeholders, including customers, investors and the public, towards organisations committed to responsible risk management. By identifying and mitigating risks, organisations can avoid financial losses and maintain stability, which is crucial for sustaining operations, meeting financial obligations and ensuring long‐term viability. Furthermore, the study identifies the best stakeholder engagement and communication practices for public health facilities. This can facilitate smoother project implementation, foster cooperation among government entities, contractors and local communities and promote a more transparent decision‐making process. Moreover, this study′s academic significance lies in its conceptual and empirical insights, which can serve as a foundation for further research. This contributes to broadening and deepening researchers′ understanding of the effectiveness of risk management practices in achieving successful outcomes in public health facilities.

### 5.4. Limitations and Future Studies

The study mainly explored risks encountered by 56 public health building projects. Thus, non‐public building projects were not considered. The study was conducted in the Dar es Salaam region, focusing on public health building projects completed and operational in the Municipal Councils of Kinondoni, Ilala, Temeke, Ubungo and Kigamboni from 2017/2018 to 2022/2023. Some projects were approved and began during the 2023/2024 financial year. Risks in building projects are numerous, leaving room for future research to explore further, probably at each building project′s phase. The current study did not categorise risks by project phase. The analysis of the risks involved descriptive analysis, which resulted in the determination of the risk heat map. This could be expanded in future research, which could use other approaches to ranking risks. Some potential approaches include the analytic hierarchy process (AHP), artificial neural network (ANN) model, fuzzy AHP and genetic algorithm–based weighting. Future studies can also focus on developing a risk assessment model or framework for building projects. The emphasis should be on using even available and already recognised tools to adopt a hybrid approach. Likewise, the study performed the regression analysis. It could have been important to calculate the variance inflation factors (VIFs) or tolerance values. However, such analysis is not included in the current study. It is recommended that future studies, when expanding the study area to other regions, should consider the calculation of the VIF. Lastly, this study examined data from 2017/2018 to 2023/2024 without comparing pre‐pandemic and post‐pandemic project phases. Data were consolidated to assess risk categories′ overall impact on project performance without time‐specific effects. The pandemic did not increase financial or procurement risks, likely because of the stability of public health construction project frameworks. Study design constraints hindered temporal variation detection. Future research should prioritise longitudinal studies to understand how external shocks like COVID‐19 affect construction risk dynamics.

## Author Contributions

**Ismail W. R. Taifa:** conceptualisation, methodology, validation, visualisation, writing – review and editing, project administration, resources, investigation, formal analysis. **Kimy Suphian Massasi:** conceptualisation, investigation, writing – original draft, methodology, validation, visualisation, software, formal analysis, data curation.

## Funding

No funding was received for this research.

## Conflicts of Interest

The authors declare no conflicts of interest.

## Supporting information


**Supporting Information** Additional supporting information can be found online in the Supporting Information section. Appendix 1: It provides the details of the closed‐ended questionnaire used to garner data from 56 public health projects in Dar es Salaam, Tanzania.

## Data Availability

The data that support the findings of this study are available from the corresponding author upon reasonable request.
