# Supplementary material for: Applied Research on the Effect of Risks on Public Health Building Projects′ Performance: Empirical Results From Tanzania
Source: ScientificWorldJournal. 2026 Jun 26;2026:2617627. doi: 10.1155/tswj/2617627 (PMC13307198; doi:10.1155/tswj/2617627)
Supplement: Supplementary file 1 — Supporting Information Additional supporting information can be found online in the Supporting Information section. Appendix 1: It provides the details of the closed‐ended questionnaire used to garner data from 56 public health projects in Dar es Salaam, Tanzania. [file TSWJ-2026-2617627-s001.docx]

**Appendix 1: Closed-Ended Questionnaire**

**SECTION I:** **Determination of risks encountered in completed public health building projects in Dar es Salaam**

Rank the impact of the following risks for the completed health projects. The scale is as follows: 1 =Very low impact, 2 = Low impact, 3= Medium impact, 4=High impact, 5=Very high impact

| **Risk category** | **Code** | **Sub-risks** | **1** | **2** | **3** | **4** | **5** |
| --- | --- | --- | --- | --- | --- | --- | --- |
| Financial risks | FR1 | Economic crisis for the financier |  |  |  |  |  |
|  | FR2 | Corruption by contractor or mid-men |  |  |  |  |  |
|  | FR3 | Increment in staff benefits |  |  |  |  |  |
|  | FR4 | Foreign currency risk- unsteady exchange rates |  |  |  |  |  |
|  | FR5 | Taxation risk- Rise in tax, customs |  |  |  |  |  |
|  | FR6 | Delay of cash flow by the client/financier |  |  |  |  |  |
|  | FR7 | Fluctuation in prices due to inflation |  |  |  |  |  |
| Management risks | MR1 | miscommunication between project stakeholders |  |  |  |  |  |
|  | MR2 | Improper project supervision |  |  |  |  |  |
|  | MR3 | Use of WBS (Work Breakdown Structure) |  |  |  |  |  |
|  | MR4 | Disorganized structures and inadequately qualified staff |  |  |  |  |  |
|  | MR5 | Shortage of skilled workers |  |  |  |  |  |
| Political risks | PR1 | Political instability |  |  |  |  |  |
|  | PR2 | Import material restrictions |  |  |  |  |  |
|  | PR3 | Failure to obtain permits |  |  |  |  |  |
|  | PR4 | Corruption of higher officials |  |  |  |  |  |
| Technological risks | TR1 | Equipment failure |  |  |  |  |  |
|  | TR2 | Obsoleteness of building equipment |  |  |  |  |  |
|  | TR3 | Lack of modern machinery |  |  |  |  |  |
| Procurement risks | RP1 | Poor quality of procured materials |  |  |  |  |  |
|  | RP2 | Shortage in the supply of water |  |  |  |  |  |
|  | RP3 | shortage in the supply of fuel and power supply |  |  |  |  |  |
|  | RP4 | Logistics delay and failure |  |  |  |  |  |
|  | RP5 | Temporary demand for an increase in materials |  |  |  |  |  |
|  | RP6 | Delay in spare-part delivery |  |  |  |  |  |
| Design risks | DR1 | Lack of innovative construction methods required |  |  |  |  |  |
|  | DR2 | errors in design drawings |  |  |  |  |  |
|  | DR3 | Inappropriate or inadequate design |  |  |  |  |  |
|  | DR4 | Design scope creep (increase of scope overtime) |  |  |  |  |  |
|  | DR5 | Survey and assessment of the site |  |  |  |  |  |
|  | DR6 | Delay in designers' response |  |  |  |  |  |
| Environmental risks | ER1 | Pollution due to construction waste |  |  |  |  |  |
|  | ER2 | Weather conditions – Precipitation, Temperature and Humidity |  |  |  |  |  |
|  | ER3 | Effects due to natural hazards/acts of god –Flood, Earthquakes, etc. |  |  |  |  |  |
| Construction risks | CR1 | Fluctuation of labour costs |  |  |  |  |  |
|  | CR2 | Shortage of labor |  |  |  |  |  |
|  | CR3 | Safety and medical outbreaks among workers |  |  |  |  |  |
|  | CR4 | Mistakes and errors of labour |  |  |  |  |  |
|  | CR5 | Non-availability of resources |  |  |  |  |  |
|  | CR6 | Quantified change of work |  |  |  |  |  |
|  | CR7 | Labour conflicts and disputes |  |  |  |  |  |
| Client or owner risks | OR1 | Unqualified owner representatives |  |  |  |  |  |
|  | OR2 | Change of project supervisors |  |  |  |  |  |
|  | OR3 | Delay or refusal of compensation to the contractor |  |  |  |  |  |
|  | OR4 | Delay in providing full possession of the site |  |  |  |  |  |
|  | OR5 | Owners’ ultra-standard expectation |  |  |  |  |  |
| Other Risks | RO1 | Theft of materials at the site |  |  |  |  |  |
|  | RO2 | Interference by the public (land grievances, strikes, etc.) |  |  |  |  |  |
|  | RO3 | Insecurity of property and site |  |  |  |  |  |
|  | RO4 | Pandemic risks (COVID-19) |  |  |  |  |  |
|  | RO5 | Accidental risk |  |  |  |  |  |

**SECTION II:** **Assessing the likelihood of risk occurrence**

Please rank the likelihood of occurrence for the following risks using a scale 1 to 5 given that; 1 – low, 2 – low medium, 3 – medium, 4 – Medium high and 5 – High.

**Note:** 1 – low (Nearly unlikely happened during the project execution and no immediate action was needed), 2 – Low-medium (Moderately unlikely happened during the project execution and minimal action was needed), 3 – Medium (Likely to occur and actions were taken to reduce or control the risk), 4 – Medium-high (More than likely to occur and management or organizers begun to mitigate), 5 – High (High probability the risk occurred; immediate action plans required)

| **Risk category** | **Code** | **Sub-risks** | **1** | **2** | **3** | **4** | **5** |
| --- | --- | --- | --- | --- | --- | --- | --- |
| Financial risks | FR1 | Economic crisis for the financier |  |  |  |  |  |
|  | FR2 | Corruption by contractor or mid-men |  |  |  |  |  |
|  | FR3 | Increment in staff benefits |  |  |  |  |  |
|  | FR4 | Foreign currency risk- unsteady exchange rates |  |  |  |  |  |
|  | FR5 | Taxation risk- Rise in tax, customs |  |  |  |  |  |
|  | FR6 | Delay of cash flow by the client/financier |  |  |  |  |  |
|  | FR7 | Fluctuation in prices due to inflation |  |  |  |  |  |
| Management risks | MR1 | miscommunication between project stakeholders |  |  |  |  |  |
|  | MR2 | Improper project supervision |  |  |  |  |  |
|  | MR3 | Use of WBS (Work Breakdown Structure) |  |  |  |  |  |
|  | MR4 | Disorganized structures and inadequately qualified staff |  |  |  |  |  |
|  | MR5 | Shortage of skilled workers |  |  |  |  |  |
| Political risks | PR1 | Political instability |  |  |  |  |  |
|  | PR2 | Import material restrictions |  |  |  |  |  |
|  | PR3 | Failure to obtain permits |  |  |  |  |  |
|  | PR4 | Corruption of higher officials |  |  |  |  |  |
| Technological risks | TR1 | Equipment failure |  |  |  |  |  |
|  | TR2 | Obsoleteness of building equipment |  |  |  |  |  |
|  | TR3 | Lack of modern machinery |  |  |  |  |  |
| Procurement risks | RP1 | Poor quality of procured materials |  |  |  |  |  |
|  | RP2 | Shortage in the supply of water |  |  |  |  |  |
|  | RP3 | shortage in the supply of fuel and power supply |  |  |  |  |  |
|  | RP4 | Logistics delay and failure |  |  |  |  |  |
|  | RP5 | Temporary demand for an increase in materials |  |  |  |  |  |
|  | RP6 | Delay in spare-part delivery |  |  |  |  |  |
| Design risks | DR1 | Lack of innovative construction methods required |  |  |  |  |  |
|  | DR2 | errors in design drawings |  |  |  |  |  |
|  | DR3 | Inappropriate or inadequate design |  |  |  |  |  |
|  | DR4 | Design scope creep (increase of scope over time) |  |  |  |  |  |
|  | DR5 | Survey and assessment of the site |  |  |  |  |  |
|  | DR6 | Delay in designers' response |  |  |  |  |  |
| Environmental risks | ER1 | Pollution due to construction waste |  |  |  |  |  |
|  | ER2 | Weather conditions – Precipitation, Temperature and Humidity |  |  |  |  |  |
|  | ER3 | Effects due to natural hazards/acts of god –Flood, Earthquakes, etc. |  |  |  |  |  |
| Construction risks | CR1 | Fluctuation of labour costs |  |  |  |  |  |
|  | CR2 | Shortage of labour |  |  |  |  |  |
|  | CR3 | Safety and medical outbreaks of workers |  |  |  |  |  |
|  | CR4 | Mistakes and errors of labour |  |  |  |  |  |
|  | CR5 | Non-availability of resources |  |  |  |  |  |
|  | CR6 | Quantified change of work |  |  |  |  |  |
|  | CR7 | Labour conflicts and disputes |  |  |  |  |  |
| Client or owner risks | OR1 | Unqualified owner representatives |  |  |  |  |  |
|  | OR2 | Change of project supervisors |  |  |  |  |  |
|  | OR3 | Delay or refusal of compensation to the contractor |  |  |  |  |  |
|  | OR4 | Delay in providing full possession of the site |  |  |  |  |  |
|  | OR5 | Owners’ ultra-standard expectation |  |  |  |  |  |
| Other Risks | RO1 | Theft of materials at site |  |  |  |  |  |
|  | RO2 | Interference by the public (land grievances, strikes, etc.) |  |  |  |  |  |
|  | RO3 | Insecurity of property and site |  |  |  |  |  |
|  | RO4 | Pandemic risks (COVID-19) |  |  |  |  |  |
|  | RO5 | Accidental risk |  |  |  |  |  |

**SECTION III: Assessing the effect of risks on the performance of completed public health building projects in Dar es Salaam**

Rate the following risk effects on the performance of the health building projects**:** 1 =Very low impact, 2 = Low impact, 3= Medium impact, 4 = high impact, 5= Very high impact.

| **Code** | **Risk influence on the project performance** | **1** | **2** | **3** | **4** | **5** |
| --- | --- | --- | --- | --- | --- | --- |
| PER1 | To what extent do the risks affect the cost of the health building projects? |  |  |  |  |  |
| PER2 | To what extent do the risks affect the schedule of the health building projects? |  |  |  |  |  |
| PER3 | To what extent do the risks affect the quality required for the health building projects? |  |  |  |  |  |

**SECTION IV: Proposing risk management practices for the public health building projects in Dar es Salaam**

Please rate the following risk management practices based on their impact on improving health-building projects in Dar es Salaam: Excellent performance= 5, Good performance= 4, Average performance= 3, Bad performance= 2, Very bad performance= 1.

| Risk management practice's category | Code | Risk management action | 1 | 2 | 3 | 4 | 5 |
| --- | --- | --- | --- | --- | --- | --- | --- |
| Avoidance or eliminate (reducing the threat or risk) | ARM1 | Removing the source of the threat |  |  |  |  |  |
|  | ARM2 | Extending the schedule prior to the start of the health-building project |  |  |  |  |  |
|  | ARM3 | Change the project management plan in terms of the project scope |  |  |  |  |  |
|  | ARM4 | Change the project management plan in terms of personnel |  |  |  |  |  |
|  | ARM5 | Change the project management plan in terms of technology |  |  |  |  |  |
| Mitigation (eliminating the probability or effect of the risk) | MRM1 | Employee or workforce training via workshops and seminars |  |  |  |  |  |
|  | MRM2 | Regular audits and updates |  |  |  |  |  |
|  | MRM3 | Incidence response plan |  |  |  |  |  |
|  | MRM4 | Use of advanced security technologies |  |  |  |  |  |
|  | MRM5 | Using fewer complex design and construction procedures |  |  |  |  |  |
|  | MRM6 | Improving the procurement procedures |  |  |  |  |  |
|  | MRM7 | Select a more stable consultant |  |  |  |  |  |
|  | MRM8 | Choosing different supplier(s) |  |  |  |  |  |
|  | MRM9 | Outlining the requirements clearly |  |  |  |  |  |
|  | MRM10 | Acquire appropriate expertise |  |  |  |  |  |
|  | MRM11 | Improve communication between stakeholders |  |  |  |  |  |
| Transfer (shifting the responsibility and effect of the threat to a third party) | TRM1 | Acquiring insurance coverage to compensate for any potential damages, thereby shifting the financial responsibility to the insurance company. |  |  |  |  |  |
|  | TRM2 | Outsourcing project activities that lack in house expertise. |  |  |  |  |  |
| Acceptance (acknowledge the risk and tolerate without executing immediate action) | ACM1 | Performing the opportunity cost (trade-off) between the benefits and disbenefits of the risks |  |  |  |  |  |
|  | ACM2 | Documenting the risk and putting aside funds in case the risk occurs (Contingency reserve to allow for time, money or resources) |  |  |  |  |  |
|  | ACM3 | Periodic examination of the risk to ensure it does not cause major effects |  |  |  |  |  |
|  | ACM4 | Creating a backup plan that would be triggered by the event |  |  |  |  |  |
